# Assessing Dietary Nutrient Adequacy and the Effect of Season—Long Training on Body Composition and Metabolic Rate in Collegiate Male Basketball Players

**DOI:** 10.3390/sports10090127

**Published:** 2022-08-24

**Authors:** Morgan M. Nishisaka, Sebastian P. Zorn, Aleksandra S. Kristo, Angelos K. Sikalidis, Scott K. Reaves

**Affiliations:** 1 Nutrition Program, Department of Food Science and Nutrition, California Polytechnic State University, San Luis Obispo, CA 93407, USA; 2 Director of Football Sports Nutrition, Stanford University, Stanford, CA 94305, USA; 3 Cal Poly Personalized Nutrition Research Group, California Polytechnic State University, San Luis Obispo, CA 93407, USA; 4 Center for Health Research, California Polytechnic State University, San Luis Obispo, CA 94307, USA

**Keywords:** athletes, collegiate basketball, body composition (BC), dietary intake, seasonal changes, resting metabolic rate (RMR), respiratory quotient (RQ)

## Abstract

The success of performance in basketball relies on both optimal body composition and nutrient intake. The purpose of this study was to examine seasonal changes in body composition (BC), resting metabolic rate (RMR) and respiratory quotient (RQ), as well as dietary intake of National Collegiate Athletic Association (NCAA) Division I (DI) male basketball players. BC, RMR and RQ were assessed during pre-season, in-season, and post-season (September, December, and March) while dietary assessment data were collected in September and February. Results of this study indicated that players received inadequate energy (*p* < 0.0001), protein (*p* < 0.001) and carbohydrate (*p* < 0.0001) relative to the recommendations for exercising individuals during the September baseline period. However, following diet analysis and consultations and relative to recommendations, athletes received adequate amounts of energy and protein during follow-up, yet intakes of carbohydrate (*p* = 0.0025) were still significantly different than recommended. Results also indicated that there was a decrease in percent body fat (%BF) during season, an increase in lean body mass (LBM) from pre- to post-season, a peak in RMR during season and an increase in RQ post-season. These findings reveal that significant metabolic and body composition changes occur in players over the season and suggest that nutritional strategies employed concomitantly may be beneficial.

## 1. Introduction

Basketball is an intermittent, high intensity sport that relies on both anaerobic and aerobic systems for energy [1]. Dietary intake is an important consideration for players, with adequate energy and macronutrient intake required for optimal body composition changes, performance, exercise recovery and overall health [2]. Without sufficient consumption of carbohydrates, there will be insufficient glycogen storage which can lead to decreases in power output and work rate, and reduced time to reaching fatigue during exercise [3]. Protein is needed in adequate amounts to provide sufficient amino acids for protein turnover, and to help preserve or gain muscle mass. Prior studies have reported that athletes tend to receive insufficient amounts of calories and carbohydrate, which can negatively impact athletic performance [4]. Therefore, additional studies are warranted to illustrate how dietary regimes in the context of season can modulate performance. 

Another factor that can affect performance of a basketball player is body composition (BC). Reduction in percent body fat (%BF) and an increase in lean body mass (LBM) has been seen to be favorable for basketball players. Studies have found that lower %BF, paired with increased LBM, has been associated with increased change of direction [5], vertical and standing long jump [6]. However, too low of %BF can have negative health implications, therefore frequently monitoring body composition is important in assessing BC changes. In addition, assessing metabolic variables such as resting metabolic rate (RMR) and respiratory quotient (RQ) aids in understanding a player’s nutrient needs, therefore allowing for more accurate and specific nutrition recommendations to be made to meet individual needs [7]. Determining an athlete’s RQ, allows for better understanding of the fuel sources being used and the athlete’s metabolic flexibility, while also examining whether an athlete is receiving adequate calories [7]. Repeatedly assessing RMR and RQ values, along with body composition in players, provides insight into understanding if changes in the nutrition recommendations are needed or if the current recommendations are in line with their needs and goals. The term “recommendation(s)” for dietary intake is used because our dietary recommendations to the athletes are based on the Dietary Reference Intake (DRI) recommendations determined by the National Academies of Science, Engineering and Medicine, previously known as the Institute of Medicine and National Academy of Sciences (see below in the “Methods” section). In the U.S., the term “prescription” has specific legal implications, and their provision is tightly regulated and can only be provided by specific certified individuals (e.g., medical doctors) and not by nutritionists or registered dietitians. That is the rationale for our use of the term “recommendation(s)” which is typically widely used in the U.S. Thus, the term “recommendation(s)” is used throughout the manuscript in the afforedescribed context.

A limited number of studies have assessed the changes in body composition over the season on collegiate male basketball players [8,9,10]. However, to the best of our knowledge, no previous studies have performed repeated measures of RMR and RQ values of the athletes over the season. Therefore, the current study aimed to examine whether there were any significant changes in body composition and metabolism in collegiate male basketball players at several time points over a season, and to assess their dietary intake compared to established recommendations for athletes. Body composition and metabolic parameters were measured at pre-season (before the beginning of the season, (i.e.: Baseline), in-season (during the season) and post-season (immediately after the conclusion of the season). Comparisons on the measured outcome variables were conducted among those time-points and against our DRI-based recommendations. 

## 2. Materials and Methods

### 2.1. Participants 

All members of the men’s basketball team (*n* = 17) voluntarily chose to participate in the study herein; however, two were excluded due to season-long injuries and two were excluded due to not all measurements being collected, resulting in 13 players completing the study. Baseline characteristics of participants are presented in Table 1. Inclusion criteria consisted of completing three dual-energy x-ray absorptiometry (DXA) scans and metabolism measurements conducted throughout the season (September, December, and March), 3-day food records and a 24-h recall. Players were also required to sign a Consent Form for Sports Nutrition Projects and Release Form for DXA scans. All participants were informed of the procedures, benefits, and risk of participating in the study and provided written informed consent prior to enrollment. All participants were made aware that they could cease their participation at any time. The study was reviewed and approved by the Institutional Review Board for Human Subjects Research of California Polytechnic State University San Luis Obispo and was in accordance with the declaration of Helsinki pertaining to human studies.

### 2.2. Procedures

During the basketball pre-season (September-October), baseline measurements were obtained. Baseline measurements consisted of anthropometrics (height, weight), BC (LBM and %BF), metabolic measurements (RMR and RQ), and dietary intake. Nutrition consultations were conducted with each player in October. Reassessment of BC and metabolic measurements were performed in December and follow up analyses of dietary intake was performed in February. Final assessments of BC and metabolism were performed post-season in March. More specifically, body composition and metabolic parameters were measured at pre-season (before the beginning of the season, (ie: Baseline), in-season (during the season) and post-season (immediately after the conclusion of the season). Comparisons on the measured outcome variables were conducted among those time-points. The dietary intake was assessed pre-season (Baseline), analyzed using ESHA software. Then, dietary intake recommendations per established DRIs were provided individually to each athlete to provide adequate nutrients per DRIs. Dietary assessment and analysis were performed again as “follow-up” in-season. Comparisons were then made among Baseline, In-season and DRI recommendations, to evaluate how close the athletes’ nutrient intake was to DRI at Baseline and after nutritional consultation in season, and thus assess dietary adequacy improvements in their diets.

### 2.3. Dietary Analyses and Nutrition Consultations

In terms of athletes’ living conditions and meal preparations, some of the athletes lived in the University housing facilities and thus consumed most of their meals on campus in dining facilities. Other athletes lived off campus and consumed most of their meals in local restaurants while also consumed meals that they themselves prepared. Dietary intake was assessed two times throughout the study. In September, participants were asked to fill out a 3-day food record (2 weekdays and 1 weekend day) to obtain baseline dietary intake details. Dietary intakes were recorded within two weeks of when the first body composition and metabolic measurements were obtained. A meeting was held with the participants to describe the proper way to fill out the food record to improve accuracy of the diet records. The information from the food records were analyzed by Food Processor III Nutrition Software version 8.6 (ESHA Nutrition Research, Salem, OR, USA). Personalized recommendations for each player were derived based on Dietary Reference Intakes (DRIs) determined by the National Academies of Science, Engineering and Medicine, previously known as the Institute of Medicine and National Academy of Sciences [11], and as described peviously [12]. Each participant’s height, weight, age, sex, and activity level were considered when estimating the requirements. Nutrition consultations with participants were given to discuss dietary intake and how it compared to nutrition recommendations for optimal health and performance, and body composition goals. During this session, three personalized ideal diets were given to participants based on their food preferences and recommendations. In February, researchs met with each participant. Participants completed a 24-h recall interview conducted by a qualified nutritionist, to assess if their dietary intake had changed since September. Another nutrition consultation session was performed with participants in February to discuss the findings from their 24-h recall compared to recommendations and baseline intake. 

### 2.4. Body Composition

Participants reported to the Nutrition and Health Assessment Lab in the Food Science and Nutrition Department of California Polytechnic State University San Luis Obispo for BC measurements in the morning of the scheduled day for measurements. BC measurements were taken using Dual Energy X-ray absorptiometry (DXA) on a Lunar iDXA (GE Healthcare, Madison, WI, USA). Each measurement was performed by the same certified technician. All procedures were performed according to GE Lunar specifications and were analyzed with enCORE software (version 11.0; GE Healthcare), as previously described [13]. The morning of each testing day, the iDXA was calibrated to verify proper function. Participants were instructed to fast for 10–12 h before each scan was taken and wear light clothing during the scan. Height and body weight were taken without shoes on, using a wall stadiometer and physician scale prior to each scan. The timeline of body composition assessment is shown below (Table 2).

### 2.5. Metabolism

Metabolism parameters measurements of each participant were completed at the Nutrition and Health Assessment Lab in the Food Science and Nutrition Department of California Polytechnic State University San Luis Obispo. Indirect calorimetry was used to determine each participant’s RMR and RQ. Each morning of testing, the metabolic cart (True One 2400- Metabolic Measurement System, ParvoMedics Inc., Sandy, UT, USA) was calibrated according to manufacture’s specifications. The testing was performed on the same morning as the DXA scans for each participant, after a 10–12 h fast. A canopy system (clear hard plastic breathing hood with drape) was placed over the participant’s head and upper body for measurements. Participants underwent testing on the metabolic cart for 25 min. The mean oxygen uptake and carbon dioxide output for each breath were measured, and the average was taken for every 15 s interval. Data from the last fifteen minutes of each session were used to calculate the RMR and RQ values.

### 2.6. Statistical Analysis

The JMP^®^ Version 16.1 (SAS Institute Inc., Cary, NC, USA, 1989–2021) was used for statistical analysis. To test for differences in 3-day dietary intake, 24-h dietary intake and recommended intake of energy and macronutrients, paired *t*-tests were used. Performance of the Shapiro–Wilk test on the dietary intake data verified normality. An exception was the meal frequency data in which the recommended number of meals per day was set at 6 for all athletes and this likely impacted normality of the data. To assess seasonal changes in body composition and metabolism measurements, a repeated measures analysis of variance (ANOVA) was used with time being the repeated measure. If significance was found for time, Tukey’s HSD post hoc analysis was done. Results were considered significant at *p* ≤ 0.05. 

## 3. Results

### 3.1. Dietary Intake 

**Figure 1:**
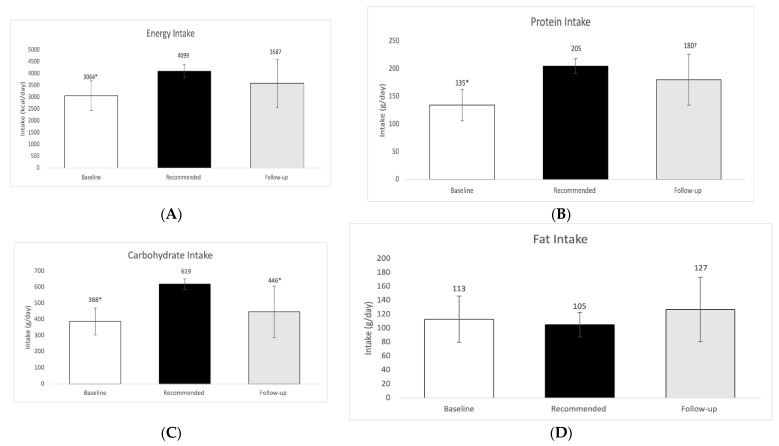
(**A**) Baseline, follow up and recommended intake of energy in kcal/day, (**B**) Baseline, follow up and recommended intake of protein in g/day; (**C**) Baseline, follow up and recommended intake of carbohydrate in g/day; (**D**) Baseline, follow up and recommended intake of fat in g/day. Results were considered significant at *p* ≤ 0.05; *n* = 13; values are presented as mean ± SD. * Denotes statistical significance between dietary intake and recommended intake. † Denotes statistical significance between baseline intake and follow up intake.

**Figure 2:**
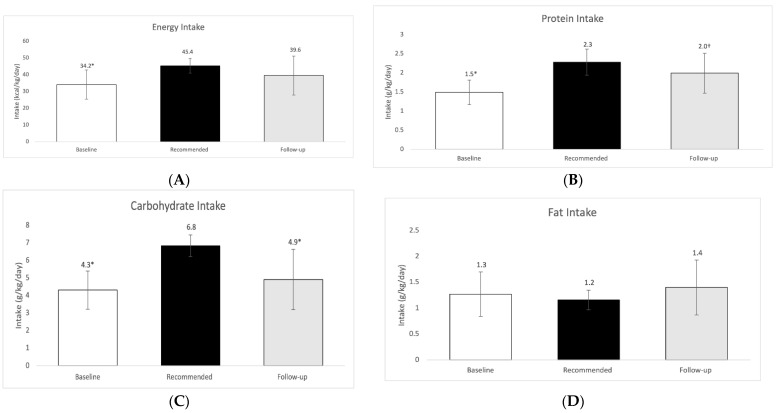
(**A**) Baseline, follow up and recommended intake of energy in kcal/kg/day, (**B**) Baseline, follow up and recommended intake of protein in g/kg/day; (**C**) Baseline, follow up and recommended intake of carbohydrate in g/kg/day; (**D**) Baseline, follow up and recommended intake of fat in g/kg/day. Results were considered significant at *p* ≤ 0.05; *n* = 13; values are presented as mean ± SD. * Denotes statistical significance between dietary intake and recommended intake. † Denotes statistical significance between baseline intake and follow up intake.

The age-, sex-, and activity-specific DRI recommendations for calories were an average of 4181 ± 298 kcal for this group. Three athletes were recommended 500 kcal less than DRI recommendations due to their goals of losing weight, therefore our average recommendations while including weight loss goals were 4099 ± 215 calories. Daily averages of dietary intake based on 3-day food records (pre-season) and 24-h recalls (post-season), as well as daily planning for energy and macronutrients can be found in Figure 1 and Figure 2.

Mean dietary intake at baseline [3064 ± 621 kcal] was significantly lower than the mean recommended intake [4099 ± 215 kcal; *p* < 0.0001]. Follow up intake showed an increase in calories consumed [3587 ± 1044 kcal], which was not different compared to the recommended intake (*p* = 0.01). Players received more calories during the time period following the initial assessment as indicated by the follow up analysis near the end of the season, although the increase was not significant (*p* = 0.1364). 

Baseline dietary protein intake [135 ± 28 g] was significantly lower than the intake recommended [205 ± 13 g; *p* < 0.0001] for athletes. An increase in protein consumption occurred during the follow up intake [180 ± 46 g] to a level that was not significantly different than the recommended level (*p* = 0.0947). The difference in protein consumption between baseline and follow up was significant [135 ± 28 g vs. 180 ± 46 g] (*p* = 0.0021) (Figure 1). We were interested in seeing what the status was when the athletes concluded the season. Obviously as the off-season period progresses it is expected to have variations in intake and most likely in energy balance since the level of activity (due to no games and less training) is significantly reduced.

Dietary carbohydrate intake during baseline [388 ± 84 g] was significantly lower than recommended intake [619 ± 33 g; *p* < 0.0001] for athletes. Although there was an increase in intake of carbohydrate during the follow up period [446 ± 160 g], intake was still significantly lower than recommended (*p* = 0.0025). The consumption of carbohydrate in the follow up did exhibit a slight but insignificant increase when compared to baseline carbohydrate intake (*p* = 0.1990) (Figure 1). 

Dietary fat intake at baseline [113 ± 33 g] was slightly higher but not statistically different than recommended intake [105 ± 18 g; *p* = 0.4556]. An increase of dietary fat occurred during the follow up [127 ± 46 g], which was insignificantly higher than recommended intake (*p* = 0.0834). The difference between baseline and follow up fat intake were not statistically different (*p* = 0.4090) (Figure 1). 

Macronutrient data were also expressed in g/kg BW/day as a means of normalization and are shown in Figure 2. The average intake at baseline was assessed to be 34.2 ± 8.8 kcal/kg of body weight, which was significantly lower than the recommended intake of 45.4 ± 4.4 kcal/kg of body weight derived by DRIs and ESHA for highly active individuals (athletes) (see methods section). The follow up dietary intake assessment during the season indicated that players consumed 39.6 ± 11.7 kcal/kg of body weight, which was insignificantly higher than at baseline. 

In our current study, players consumed 1.5 ± 0.3 g/kg of body weight of protein during baseline which was significantly lower than the recommended amounts in the study, of 2.3 ± 0.3 g/kg. Players consumed 2.0 ± 0.5 g/kg during follow up assessment, which was insignificantly different than recommended intake. The difference in protein consumption between baseline and follow up was significant [1.5 ± 0.3 g/kg vs. 2.0 ± 0.5 g/kg (*p* = 0.0021)] (Figure 2). The present study also found that carbohydrate intake at baseline (4.3 ± 1.1 g/kg of body weight) was significantly lower than the recommendation and remained significantly lower at the dietary intake follow up assessment. 

Unlike the previously discussed macronutrients, fat intake was not significantly different at baseline compared to recommendations and increased, although not significantly, during the follow up. The follow up fat intake was insignificantly different compared to recommended intake. Follow up intake was 1.4 ± 0.5 g/kg of body weight, which is 120% of the recommended intake of 1.2 ± 0.2 g/kg of body weight. 

**Figure 3:**
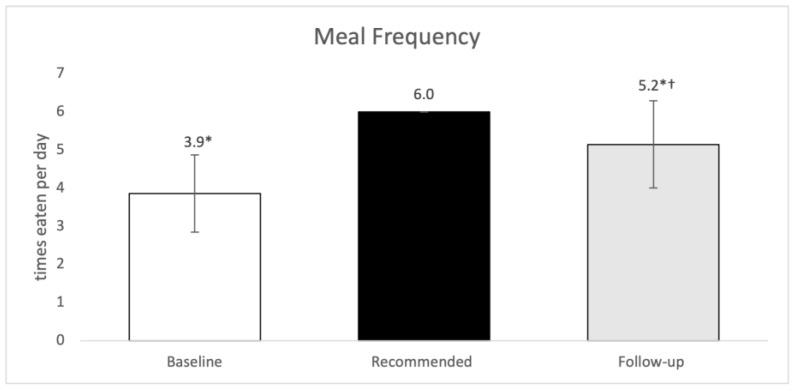
Baseline, follow up and recommended frequency in meals eaten per day. Results were considered significant at *p* ≤ 0.05; *n* = 13; values are presented as mean ± SD. * Denotes statistical significance between dietary intake and recommended intake. † Denotes significance between baseline intake and follow up intake.

The frequency of meal consumption per day at baseline [3.9 ± 1.0 times per day] were significantly below the recommended amount [6.0 times per day; *p* < 0.0001] for athletes. The follow up showed a significant increase in the number of meals that the players consumed [5.2 ± 1.1 times eaten per day; *p* = 0.0008]. Figure 3 shows average meal frequency and recommendations.

### 3.2. Body Composition

DXA scans were performed at three time points. Table 3 presents the body composition data before, during and post-season.

No significant changes were seen throughout the season in players’ weights (*p* = 0.899). %BF and LBM changes were significant at specific time points during the season (*p* = 0.0018; *p* = 0.0436) as shown in Table 4. Following pre-season, a decrease in %BF was observed and remained constant through post-season [mean difference; 95% CI: 1.092; 0.34, 1.84]. LBM increased significantly at post-season compared to pre-season [mean difference, 95%CI: 1.127; 0.01, 2.24]. There was not a significant difference found in LBM between in-season and the other two time points (pre- and post-season). 

### 3.3. Metabolism

Each athlete’s metabolic data were assessed at three time points that were the same as the DXA scans. Table 4 presents the RMR and RQ data. 

There were significant differences observed in the RMR between time points during the season (*p* = 0.0069). The RMR was significantly higher in-season compared to pre-season [mean, difference, 95% CI: 161.08; 41.63, 80.52] and post-season [mean differece; 95% CI: 122.06; 2.62, 241.50]. The post-season RQ was signficantly higher as compared to the pre- and in-season timepoints [*p* < 0.001; mean difference, 95% CI: 0.067, 0.027, 0.11]. 

## 4. Discussion

To the best of our knowledge, no previous research has investigated seasonal changes in body composition and metabolism variables, as well as the dietary intake, in NCAA Division I male basketball players within the same study. Therefore, the purpose of this study was to examine these factors while aiming to provide interesting and helpful insight for athletes, coaches and trainers of this popular collegiate sport. Three major findings are reported: (1) male basketball players’ pre-season energy, protein, and carbohydrate intake were significantly lower compared to recommended intake values. During the follow up intake assessment during the season, players demonstrated receiving adequate energy and protein, yet carbohydrate intake was still significantly lower than recommended. (2) Body composition changes included that %BF exhibited a significant decrease by the in-season time point, and LBM was highest at the post-season time point. (3) RMR was significantly elevated at the in-season time point, and the RQ was highest at the post-season time point. 

### 4.1. Dietary Intake 

At baseline the energy and macronutrient intake, with the exeption of fat, (Figure 1 and Figure 2) of athletes were found significantly lower than DRI amounts as per the dietary intake analysis. Assessment results were shared with athletes during consultative educational meetings at which time information and personalized example dietary intake plans were given to each athlete. At the follow up dietary analysis, there was evidence that protein intake had significantly increased relative to baseline values. Dietary intake data expressed as kcal/kg body weight indicated that the average intake at baseline (34.2 ± 8.8 kcal/kg body weight) was significantly lower than the recommended intake (45.4 ± 4.4 kcal/kg body weight) derived by DRIs and ESHA for highly active individuals (athletes) (see methods section). Follow up dietary intake assessment during the season indicated that players consumed somewhat higher amounts of kcals compared to baseline albeit not quite significantly, in line with the result of previous studies [14,15]. One study assessing the dietary intake of collegiate female basketball players (*n* = 10) throughout the season found that the players were not receiving as much energy during pre-season (1925 ± 466 kcal/day) but their intake increased significantly at the end of the season (2326 ± 782 kcal/day) [14]. Perhaps pre-season low energy intake in basketball players could reflect their decreased appetite and energy demands compared to in-season. Another contributing factor for the inadequate intakes, could be concerns of weight gain and body fat increases, a trend commonly observed among various young athletes [16]. This concern could possibly suppress food intake even in the presence of increased appetite during the season. 

In our current study, players consumed significantly less protein during baseline than our recommended amounts in the study. The recommended intake 2.3 ± 0.3 g/kg body weight) in our study herein was above the 1.5–2.0 g/kg recommendation for strength athletes [17]. The players exhibited a hypocaloric state during baseline data collection, therefore the increase in protein intake was recommended, in order to support preservation of lean body mass [18]. Players consumed 2.0 ± 0.5 g/kg during follow up assessment, which was insignificantly different than recommended intake. Perhaps, this increase in dietary protein intake is one factor that helped players gain lean mass throughout the season, although we cannot be certain due to the the difference in time points of data collection. Multiple studies involving basketball players have shown that players tend to consume amounts of protein within the recommended ranges of 1.2–2.0 g/kg when assessed via dietary records [19,20]. 

The present study also found that carbohydrate intake at baseline was significantly lower than the recommendation and remained significantly lower at the dietary intake follow up assessment. During baseline, players in this study consumed 4.3 ± 1.1 g/kg of body weight carbohydrate. A study looking at the dietary habit of elite Spanish basketball players (*n* = 55) showed that carbohydrate consumption was 4.6 ± 1.7 g/kg body weight [20], similar to the findings of our study. Other studies have also found carbohydrate intake to be similar in agreement with our study [15,21]. Chronic low carbohydrate intake, below the recommendations of 6–10 g/kg of body weight for endurance athletes, may result in the basketball players having inadequate glycogen stores before play, which in turn may decrease performance and time to fatigue [2]. 

An exception to the previously discussed macronutrients was that fat intake was not significantly different at baseline compared to recommendations and increased, although not significantly, during the follow up. The follow up fat intake was insignificantly higher as compared to recommended intake. Findings from other studies have also shown that fat intake tends to be significantly higher than recommended [19,20]. For example, Short and Short (1983) found that average fat intake of collegiate male basketball players ranged from 196 to 254 g over the course of 4 years [19]. The demands of balancing training and school schedules can cause college athletes to gravitate towards readily available foods higher in fat [22], an observation in our study as well, and this may at least partially explain our data.

In the present study, the average meal frequency at baseline was 3.9 ± 1.0 times per day, and this seems to be consistent with previous studies. One study involving female college athletes found similar results to our study, where the mean dietary intake reported was 3.8 ± 0.9 meals per day at baseline [15]. Another study involving elite basketball players, showed that 66% of the players did not consume at least 3 meals a day [23]. Findings from our current study show that during the follow up, assessment of meal frequencies indicated that frequency had significantly increased to 5.2 ± 1.1 times eating per day, perhaps in relation to a more structured daily schedule and/or increasd appetitte due to training demands, yet this was still significantly less than recommended. Inconsistent intake of nutrients throughout the day can decrease the players’ ability to adhere to nutrient timing, which can subsequently confer consequences on a player’s performance and recovery [2]. Improvements in dietary parameters after the consultation implies lack of nutrition knowledge in athletes, previously reported [24], as well as efforts of compliance to recommendations, thus emphasizing the need for personalized nutrition advice in athletes.

### 4.2. Body Composition

The findings from this study indicated that %BF and LBM significantly changed at time points during the season, which is not surprising given the increased physical demands of practice and play. %BF was higher during pre-season and decreased significantly during in-season and remained decreased for post-season. Significant seasonal %BF changes among basketball players are inconsistent in previous studies. One study examining BC changes in collegiate male basketball players, found that the BF significantly decreased by 2.3 kg from pre-season to post-season. Other studies reported similar findings to our study, where there was a decrease in FM and %BF when comparing pre-season to post-season measurements [10,23,25,26,27,28]. On the contrary, two other studies in male basketball players showed no significant changes for %BF throughout the season [8,9]. Lower %BF during the season may be desired, as it has been shown to improve performance, allowing for an increase in vertical and standing long jump, when paired with increases in LBM [6].

Our study also found that LBM increased significantly from pre-season to post-season, which is similar to the findings of other studies [10,26,27]. Aligning with the findings from our study, the same study that found a decrease in FM in collegiate male basketball players also documented a significant increase of 1.6 kg in FFM from pre-season to post-season [10]. Unlike the findings from our study, Fields et al. (2018) reportedly found no changes to the LBM of collegiate male basketball players throughout the season [9]. The studies that did not align with our findings used other forms of measurement for BC (i.e., Skinfolds and air displacement plethysmography), which may explain the inconsistency in their findings relative to those of our study. Therefore, it is important to consider the method that was used to gather BC data when comparing values/studies. 

### 4.3. Metabolism

To the best of our knowledge, other studies have not examined RMR and RQ at pre-season and post-season in collegiate male basketball players. In the current study, RMR significantly increased at the in-season time point compared to pre- and post-season time points, in agreement with the observed increase in LBM in our participants. A study involving male elite rugby players found that RMR increased for three days after a game, compared to the day before the game, yet RMR did not change after training days [29]. These obervations may potentially serve as an explanation as to why there was an increase in RMR during season compared to post-season in our study. 

Regarding the RQ data, there was a significant increase from in-season to post-season. While in pre-season and in-season, players had an RQ value of less than 0.7, which is an indication that players were undereating similarly to previous observations [30]. The increase in RQ post-season serves as an indication that the players were receiving adequate amounts of calories to meet their energy needs. 

### 4.4. Strengths, Limitations and Future Outlook

To the best of our knowledge this study is the first of its kind to investigate seasonal BC and metabolism changes in NCAA Division I male basketball players, while also assessing their dietary intake. This study used DXA, considered by many to be the gold standard when assessing body composition [31,32]. Perhaps a limitation of this study was the small sample size of as we worked with 15 players and 13 completed the study and were included in data anlyses. However, this group of participants essentially comprised the entire basketball team and in this sense, it constituted an entire unit. Another potential limitation of this study may be the different methods of dietary intake assessments used; the 24-h recall during the follow up period only gives a snapshot of one day of intake, whereas the 3-day food record that was used for baseline collection is an average of various days throughout the week. The variances in the 24-h recalls are greater when compared to the 3-day food records as indicated by some evidence [33]. Using a 3-day food record at several time points in a future study may provide more comprehensive information regarding food intake. A challenge in working with Collegiate athletes is their demanding schedule and availability. That constituted a limitation in terms of the frequency their dietary intake was assessed.

## 5. Conclusions

The objective of our study was to compare the athletes at three different time points in a competitive season for body composition and metabolic parameters. We also evaluated athletes’ adherence to a recommended nutrition plan. In conclusion, we found that NCAA Division I male basketball players appeared to consume significantly less than optimal amounts of food compared to recommended amounts early in the season (baseline). Significant changes in body composition occurred over the course of the season compared to baseline. In addition, changes in metabolic parameters (RMR, RQ) were observed over the course of the season. This study may be the first to address the dietary, body composition and metabolism changes in NCAA Division I male basketball players, future research needs to be performed to fully understand the demands and effects of the season and how dietary strategies including a personalized nutrition approach may promote positive effects. The changes to dietary intake should be monitored in athletes to ensure adequate energy and macronutrient intakes to optimize BC, performance and health. Regular BC and metabolism measurements should be periodically conducted, as well as the assessment of dietary intake, to ensure proper dietary recommendations are being given to athletes to meet their needs and goals.

## Figures and Tables

**Table 1:** Baseline Characteristics of Participants (Basketball Players).

Number of Players	13
Age	20.45 ± 1.20
Height (cm)	189.86 ± 7.55
Weight (kg)	91.33 ± 11.62

*n* = 13; values are presented as mean ± SD.

**Table 2:** Timeline of Body Composition Assessments via DXA.

Scheme of Body Composition Assessments			
	September	December	March
DXA scans	X	X	X

**Table 3:** Body Composition changes throughout the season.

Measure	Pre-Season (Baseline)	In-Season	Post-Season
Weight (kg)	91.33 ± 11.62 ^a^	91.18 ± 10.53 ^a^	91.46 ± 10.77 ^a^
Lean Body Mass (kg)	74.63 ± 8.87 ^a^	75.53 ± 8.59 ^a,b^	75.75 ± 9.26 ^b^
% BF	13.48 ± 3.60 ^a^	12.39 ± 3.60 ^b^	12.46 ± 3.54 ^b^

Different superscript letters denote statistical significance. Results were considered significant at *p* ≤ 0.05; *n* = 13; values are presented as mean ± SD.

**Table 4:** Measurements of RMR and RQ before, during and postseason.

Measure	Pre-Season (Baseline)	In-Season	Post-Season
RMR (kcal/day)	2329.93± 232.85 ^a^	2491.01± 215.15 ^b^	2368.95 ± 264.13 ^a^
RQ	0.696 ± 0.04 ^a^	0.676 ± 0.03 ^a^	0.763 ± 0.06 ^b^

Different superscript letters denote statistical significance. Results were considered significant at *p* ≤ 0.05; *n* = 13; values are presented as mean ± SD.

## Data Availability

Data is available upon request from corresponding author.
